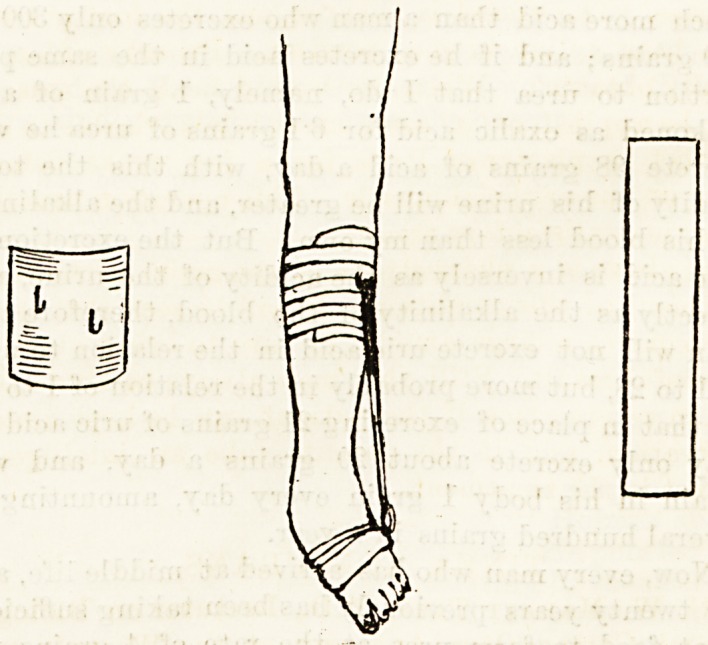# Treatment of Club-Foot

**Published:** 1894-04-21

**Authors:** Arnold W. W. Lea

**Affiliations:** Late Senior Medical Officer to the Hospital for Sick Children, Pendlebury


### TREATMENT OF CLUB-FOOT.

By Arnold W. W. Lea, M.B., B.S.Lond., F.R.C.S.Eng. Late Senior Medical Officer to the Hospital for Sick Children, Pendlebury.

There are several deformities of the foot grouped under this heading. Talipes equino varus is, however, the one we are most frequently called upon to treat. This is usually congenital, though a similar deformity occasionally results from infantile paralysis. The treatment of these cases requires a large amount of care and patience both from the surgeon and the parents.

The treatment of a congenital case cannot be commenced too early, as a great deal of improvement can be effected during the first few months of life, when the structures are soft and yielding.

The foot should be frequently moulded into good position by the surgeon, and during the intervals splints should be carefully applied. We must remember, however, that the skin of a young infant is very tender, and liable to pressure sores if great care be not taken. If the child is seen and splints can be applied successfully during the first few months, operation is frequently not required, providing the foot can be brought into good position by moulding.

The splint which is used at the Pendlebury Hospital is the straight tin splint, as shown in the diagram. This is padded and bandaged along the outer side of the leg and foot in the varus position; the splint is now bent slightly outwards carrying the foot with it and partially correcting the varus. No attempt is made at first to correct the equinus.

This is frequently reapplied until the varus is completely corrected. Afterwards, the equinus is gradually corrected by the same splint applied to the back of the leg and turned up on the sole. The heel is thus gradually brought down to a right angle. When the deformity is thus quite corrected an artificial muscle is applied, after Barwell's plan. A piece of strapping or webbing is carried round the foot from within outwards under the sole. To the end of this a piece of india-rubber cord is fixed. This passes up from the outer side of the foot up the leg. On the outer side of the leg a piece of tin is fixed by strapping, which has two hooks soldered into it. Over one of these the loop of cord is fastened. In this way continuous gentle traction of the foot is maintained. Instead of the tin plate a leather apparatus may be fixed above and below the knee, to which the cord may be attached. The duration of the treatment of these cases is very variable, and depends to a great extent upon the amount of care which can be bestowed. If the muscle is not worn for some time there is a great tendency to relapse.

Of course, in many cases the above means have to be supplemented by operative measures. If the foot cannot be brought into good position, and the child is some months old, tenotomy will probably be required.

Great benefit is often derived from the subcutaneous division of the tense structures on the inner border of the foot, notably the plantar fascia and the ligaments. The tenotome is entered over the astragalo-scaphoid joint, the structures are divided until the foot comes into position. In some cases it is necessary to cut all the soft structures down to the astragalo-scaphoid joint before the foot will yield; in this way the powerful calcaneo-scaphoid ligament is divided. This operation usually gives very good results. The tendo-achillis should also be divided in these cases. Sometimes it may also be necessary to perform tenotomy of the tibialis anticus tendon in addition. The foot may generally be put up at once in the corrected position, and in a short time the artificial muscle may be applied as above.

In severe and neglected cases of talipes coming under observation when the children are several years old, resection of some of the bones of the tarsus will be required in order to bring the foot into good position. An incision is made along the outer border of the foot, the peronei tendons are drawn aside and the bones exposed. A wedge-shaped piece of bone is now removed by a chisel. The cuboid is usually taken away and portions of the adjacent bones, usually the os calcis, astragalus, scaphoid and cuneiforms, the amount to be taken away varying with the severity of the case. The wound is sutured, and the foot put up on a back splint in good position. Primary union is usually obtained. When the wound heals the muscle may be applied, or in some cases it is advisable to fix the foot in plaster of Paris for a few weeks before fixing on the artificial muscle. This operation usually gives very satisfactory results.

The above is a short sketch of the main methods of treatment of this condition. It will be seen that each case requires a large amount of care to bring about a successful result. Still this may always be obtained, even in severe cases, if the patient can be kept under observation, and if the parents take an intelligent interest in the treatment.

## Figures and Tables

**Figure f1:**